# A study of nitrogen dioxide (NO_2_) periodicity over the United Arab Emirates using wavelet analysis

**DOI:** 10.1038/s41598-022-21937-3

**Published:** 2022-10-28

**Authors:** Aishah Al Yammahi, Zeyar Aung

**Affiliations:** Khalifa University of Science and Technology, Abu Dhabi, United Arab Emirates

**Keywords:** Climate sciences, Environmental sciences

## Abstract

NO_2_ and nitric oxide (NO) are the most reactive gases in the atmosphere. The interaction of NO_x_ molecules with oxygen, water and other chemicals leads to the formation of acid rain. The presence of NO_2_ in the air affects human health and forms a photochemical smog. In this study, we utilize wavelet analysis, namely, the Morlet wavelet, which is a type of continuous wavelet transform, to conduct a spectral analysis of the periodicity of nitrogen dioxide (NO_2_). The study is conducted using data from 14 weather stations located in diverse geographic areas of the United Arab Emirates (UAE) over a period of two years (2019 and 2020). We explain and relate the significance of human activities to the concentration level of NO_2_, particularly considering the effect of the COVID-19 lockdown to the periodicity of NO_2_. The results show that NO_2_ concentrations in desert areas such as Liwa and Al Quaa were unaffected by the lockdown period (April–July 2020) resulting from the COVID-19 pandemic. The other stations in the urban areas of Abu Dhabi city, Al Dhafra and Al Ain, showed a reduction in NO_2_ during the lockdown. NO_2_ is more highly concentrated during winter seasons than during other seasons. The periodicity of NO_2_ lasted from a few days up to 16 days in most regions. However, some stations located in the Al Dhafra region, such as Al Ruwais and the Gayathi School stations, exhibited a longer period of more than 32 days with a 0.05 significance test. In the Abu Dhabi region, NO_2_ lasted between 64 and 128 days at the Al Mafraq station. The correlation between the NO_2_ concentration across several ground stations was studied using wavelet coherence.

## Introduction

Wavelet transform is a statistical method based on time series that is used in various fields, such as economics, climatology, astronomy, and medical image processing^1–4^. Climate variability can be studied by using wavelet analysis to track patterns, cycles and periodicity^5^.

Climatic time series data are either stationary or nonstationary. The Fourier transform is usually used for stationary time series data. However, wavelet transform is used to track the periodicity and variability of nonstationary climatic and meteorological parameters, such as temperature and precipitation, and atmospheric composition, such as sodium, sulfate concentrations, carbon dioxide (CO2), and other greenhouse gases (GHGs)^1,6–9^. Wavelet analyses are also used in other domains, such as studying the variability in phytoplankton seasonal and annual periodicity^10^ and identifying the monsoon drought index period over India^11^.

Some GHGs, such as nitrogen dioxide (NO_2_), sulfur dioxide (SO_2_) and carbon monoxide (CO), were studied over China^12^ and Poland^13^ during the lockdown that resulted from the COVID-19 pandemic. Some researchers have focused on studying the variability of nitrogen dioxide (NO_2_) and sulfur dioxide (SO_2_) specifically more than other air pollution components because of their serious impacts on human health. Additionally, the emissions of these gases increase proportionally with the growth of human industrial activities^7,12–14^.

Recently, in^7^, the spatiotemporal fluctuation of the NO2 tropospheric column concentration from 1996 to 2017 in Italy was studied based on human activities in terms of gross domestic product (GDP). The study related GDP, an indicator of economic performance, to its effect on air quality and NO_2_-induced pollution. In addition to human activities, the study considered the nature of NO_2_, as it is a reactive trace gas; its emissions are changeable during the daytime. The lifetime of NO_2_ varies in different seasons from a few hours in summer to a day during winter. It also lasts for more days if it reaches the height of the boundary layer between the troposphere and stratosphere. The wavelet power spectrum classified NO_2_ into three periods: 1999–2007, 2007–2013 and 2013–2016. It was reported that the reduction in NO_2_ presented in the second period was due to the global financial crisis in 2008^7^.

The changes in air quality based on the daily mean concentrations of five atmospheric components, PM2.5, PM10, SO_2_, NO_2_, and CO, were studied in Shanghai, China^12^, using descriptive statistics during the lockdown that resulted from COVID-19. The data were obtained from the China National Environmental Monitoring Center of Ministry of Ecology and Environment for 3 months (January–March) over 4 years from 2017 to 2020. The results show that daily concentrations of PM2.5, PM10, SO_2_, NO_2_, and CO during the lockdown period were reduced by 9%, 77%, 31.3%, 60.4%, and 3%, respectively^12^.

A similar study was performed in Poland^13^ during the COVID-19 pandemic regarding the impact of four pollutants (PM2.5, PM10, NO_2_ and SO_2_) during the lockdown. The data were obtained from satellite (MODIS & TROPOMI) and ground stations from January to 25 April 2020 and aligned with the same time period in 2019 to study the absolute differences. The study period was divided into groups: before lockdown, during lockdown and after lockdown. The study used simple mathematics and statistics, and the results showed a reduction in the concentrations of PM2.5, PM10, NO_2_ and SO_2_^13^.

The Haar wavelet was used to study NO_2_ and SO_2_ in Uttar Pradesh, which is located in northern India, for two time periods before the lockdown (01-01-2018 to 31-12-2019) and two time periods after the lockdown (01-06-2020 to 31-07-2020). The data are provided from the website of the Central Pollution Control Board. The results show that the lockdown had a positive effect on the environment because it caused a reduction in the average concentrations of NO_2_ and SO_2_^14^.

Wavelet coherence is used to address the correlation, either negative or positive, between variables such as temperature and CO_2_ over the time period, as done in^15^. Additionally, multiple wavelet coherence (MWC) is used to study the correlation between more than two variables, using one variable as a dependent variable and the others as independent variables. MWC has been applied to soil water content using several factors, including depth to CaCO_3_ layer, soil organic carbon and sand content^16^. Generally, wavelet coherence has some unique features when compared to traditional time series methods. It is able to compare the negative and positive correlations between variables in one scalogram in one time period^17^.

The aim of this research is to study the periodicity of NO_2_ at 14 ground stations in the UAE during the two years of 2019 to 2020 using Morlet wavelet analysis^18^. We explain and relate the effect of the COVID-19 lockdown to the periodicity of NO_2_, as NO_2_ is a source of air pollution and the pandemic occurred within the period of interest for this study. Previous studies used simple mathematics and statistics to address the concentration of NO_2_ during the lockdown. However, in this study, we use a more advanced method, wavelets. Wavelet coherence analysis has been used to study the relation between NO_2_ concentrations across some ground stations^19^.

## Data and methods

### NO_2_ concentration data in the UAE

**Figure 1:**
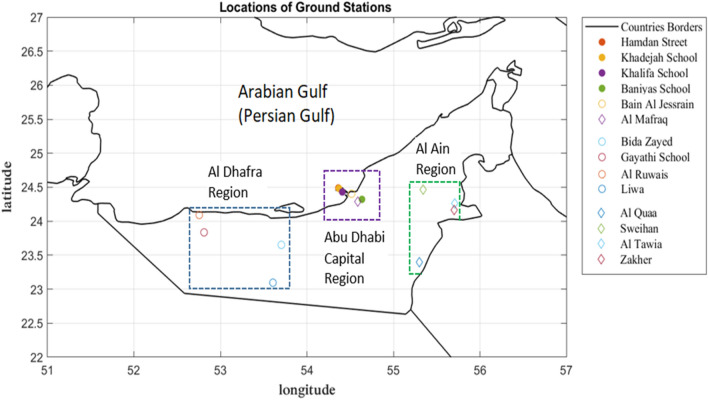
The locations of 14 ground stations on the map of the UAE (note: stations 1–3 are located relatively close to each other in Abu Dhabi City).

The United Arab Emirates (UAE) is located between latitudes 22.5° N to 26.5° N and longitudes 51.6° E to 56.5° E. The ground station data for 14 sites on the Abu Dhabi Emirates were provided by the Environment Agency—Abu Dhabi for 2 years: 2019 and 2020. The fourteen ground station sites are distributed in the Abu Dhabi Capital region, Al Dhafra region and Al Ain region, as shown in Table 1 and Fig. 1, to monitor the atmospheric composition, which affects air quality. The ground station locations are classified into different categories: (i) urban, (ii) suburban, and (iii) rural. The concentration of NO_2_ measured from the ground stations is provided hourly in units of µg/m^3^. This study uses the daily average NO_2_ concentration. Then, the outliers are identified and clipped to three standard deviations from the mean (covering 99.7% of the original data)^20^.

**Table 1:** Ground station network in the Abu Dhabi, Al Dhafra, and Al Ain regions.

Station ID	Name	Station type (land use of surrounding area)	Region
1	Hamdan Street	Urban traffic	Abu Dhabi Capital Region
2	Khadejah School	Urban background	Abu Dhabi Capital Region
3	Khalifa School	Suburban background	Abu Dhabi Capital Region
4	Bani Yas School	Suburban background	Abu Dhabi Capital Region
5	Bain Al Jessrain	Suburban background	Abu Dhabi Capital Region
6	Al Mafraq	Suburban industrial	Abu Dhabi Capital Region
7	Bida Zayed	Suburban background	Al Dhafra Region
8	Gayathi School	Suburban background	Al Dhafra Region
9	Al Ruwais	Suburban industrial	Al Dhafra Region
10	Liwa	Rural background	Al Dhafra Region
11	Al Quaa	Rural background	Al Ain Region
12	Sweihan	Suburban background	Al Ain Region
13	Al Tawia	Suburban background	Al Ain Region
14	Zakher	Urban background	Al Ain Region

**Figure 2:**
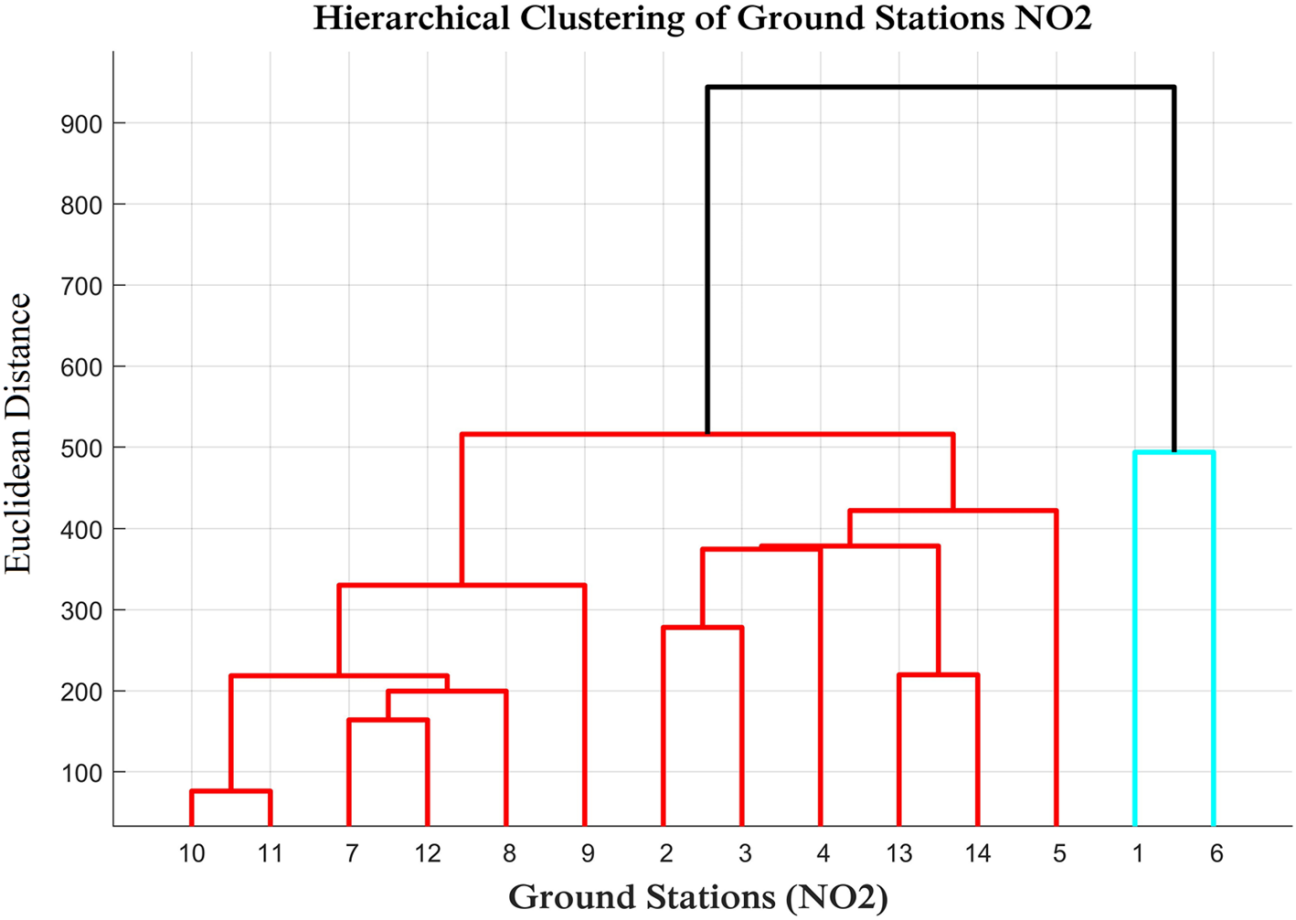
Hierarchical clustering of the ground stations based on their NO_2_ concentration profiles over the period of 2019–2020. The lower the Euclidean distance is, the more similar the NO_2_ concentration profiles of the two stations are.

To elucidate the relationship between the NO_2_ concentration level and the land use of the surrounding area of a ground station, we conducted hierarchical clustering using Ward’s linkage criterion^21^ of the ground stations’ NO_2_ concentration profiles over the period of 2019–2020. The clustering results are depicted in Fig. 2, in which the similarities of the NO_2_ concentrations across the ground stations are presented. The lower the Euclidean distance is, the more similar the NO_2_ concentration profiles of the two stations are. It is observed that land use is related to NO_2_ concentrations at many stations, regardless of their geographical distance.

It was observed that the NO_2_ concentration profiles of Liwa (Station 10) and Al Quaa (Station 11), both located in rural areas, are very similar (with the lowest Euclidean distance). The profiles of Bida Zayed (Station 7), Sweihan (Station 12), and the Gayathi School (Station 8) are found to be clustered together, as they are all located in small towns (suburban background) with populations of approximately 29.1 thousand, 5.4 thousand, and 14.0 thousand, respectively. The concentration of NO2 in all schools (Stations 2, 3, and 4) that lie in the Abu Dhabi City Region (a large city with 1.5 million population) are in one cluster. Hamdan Street (Station 1, urban traffic in Abu Dhabi) and Al Mafraq (Station 6, suburban industrial in Abu Dhabi) also fall into one cluster even though the Euclidean distance between them is quite high. Located in downtown Abu Dhabi and an industrial area, these two stations exhibit the highest overall NO_2_ concentration levels.

### Morlet wavelet analysis

The Fourier transform translates signals from the temporal domain into the frequency domain, yet it does not localize the time of the frequency components. It is useful for studying stationary signals. Therefore, the short-time Fourier transform (STFT), which uses windowing to afford time localization to Fourier’s frequency, is developed to overcome the limitation of localizing the time. The STFT treats each segment of signals as a discrete portion of a stationary process by using a finite window function to specify the time windows and compare the signal with the known windowed function. There are many types of windowing functions, such as Gaussian curves, rectangular window functions, and triangular window functions. The fixed size of the windows creates a limitation in the resolutions for both time and frequency. Therefore, a high time resolution results in low frequency resolution, and vice versa; thus, an empirical test is needed for various window lengths to determine the most relevant among them^6,22^.

There is another type of windowing function called wavelet. There are two wavelet transform methods: discrete and continuous. Discrete wavelet transforms (DWT) are used for noise reduction and data comparison. Continuous wavelet transforms (CWTs) are used for feature extraction. There are three main mother functions of CWT: Morlet, Paul, and Mexican Hat. Morlet wavelet analysis is extensively used in climatic studies in various time and frequency domains to extract the features of the pattern and periodicity of a certain variable^18,23–25^. The significance levels and confidence intervals are also considered in the Morlet wavelet power spectrum, which identifies the periodicity in climatic components^23^. Wavelet transform has advantages over Fourier transform as it localizes the time of the spectral characteristics. The time–frequency window of the wavelets is not fixed as in the STFT. Multiscale windows in wavelet give higher resolution by including the high- and low-frequency cycles^26,27^.

The continuous wavelet transform (CWT) is used to study a one-dimensional signal and decompose it into scale $(a)$ and time (τ). The CWT has the advantage of localizing the spectral components along the time domain, which helps in recognizing the changes in nonstationary signals even if the signal is short-lived, as the scale $a$ can compress or extend the function to pass over the signal. The wavelet transform decomposes a signal and expresses it as a sum of wavelets, and the continuous wavelet transform (CWT) signal in the time domain $x(t)$ is defined as in Eq. (1).

1 $${\mathrm{C}}_{\uppsi }^{x}\left(a,\tau \right)=\frac{1}{\sqrt{a}} {\int }_{-\infty }^{+\infty }{x\left(t\right)\uppsi }^{*}\left(\frac{t-\tau }{a}\right)dt$$

where (*) symbolizes the complex conjugate of the wavelet function, $a$ is the scaling parameter, and τ is the location parameter that slides (transform) the wavelet over the time domain. The transform acts as a convolution function, which is an integration of the product of the signal $x(t)$ and sliding wavelet function ψ. The convolution measures the similarity between the signal and the shifting function from the overlap between them. The shape of the wavelet function can be modified by the scaling parameter $a$, which stretches and compresses the mother wavelet into its daughter wavelets. It also changes the temporal duration of the windowing function by altering the window width and thus captures the frequency features. The energy of the mother wavelet and the energy of the scaled wavelet remain the same by normalizing them with a factor $1/\surd a$ . Therefore, the coefficient ${\mathrm{C}}_{\uppsi }^{x}\left(a,\tau \right)$ and its power ${|{\mathrm{C}}_{\uppsi }^{x}\left(a,\tau \right)|}^{2}$ are comparable. All the possible scales are applied in CWT; it is independent of the transform, unlike STFT.

Different types of localized waveforms can be applied if they satisfy the three mathematical criteria. The first is that it must have finite energy, which characterizes the signal. The second is the admissibility condition to ensure that the CWT can be inverted; it can reconstruct the original signal by applying the inverse of the CWT. This implies that the wavelet has a zero mean and no zero frequency. If $\widehat{\uppsi }\left(f\right)$ is a Fourier transform as in Eq. (2), then the admissibility constant ${C}_{g}$ is finite, as illustrated in Eq. (3); it is dependent on the type of wavelet chosen. The admissibility criteria were achieved by using the frequency of 6.0^6,14,28,29^.

2 $$\widehat{\uppsi }\left(f\right)={\int }_{-\infty }^{+\infty }\uppsi (\mathrm{t}){e}^{-i\left(2\pi f\right)t}dt$$

3 $${C}_{g} ={\int }_{0}^{\infty }\frac{{\left|\widehat{\uppsi } \left(f\right)\right|}^{2}}{f}df<\infty$$

The third criterion considers the real part of the complex wavelet used to compute the squared magnitude and remove the negative frequencies. This creates a scalogram that is defined as a two-dimensional wavelet energy density function, as shown in Eq. (4). The energy distribution is demonstrated in a three-dimensional graph (scalogram) with a scale range (y-axis), time domain (x-axis), and energy in a color gradient scale. The scale is inversely proportional to the frequency and directly proportional to the period, so the y-axis can be represented by the period range. Different forms of the scalogram equation can be considered if the function varies from Eq. (5) by a constant multiplicative factor such as (1/${C}_{g}$) or (1/${C}_{g}{f}_{c}$), where ${f}_{c}$ is the characteristic frequency of the wavelet function. The total energy in the signal can be recovered by integrating the scalogram over the wavelet parameters $a$ and τ using the admissibility constant, as shown in Eq. (3). The Morlet wavelet is characterized by ${\omega }_{0}$ > 5.0^6,14,28^.

4 $$\mathrm{E}\left(a,\tau \right) {=\left|{\mathrm{C}}_{\uppsi }^{x}\left(a,\tau \right)\right|}^{2}$$

5 $$E=\frac{1}{{C}_{g}}{\int }_{-\infty }^{+\infty }{\int }_{0}^{+\infty }\frac{{\left|{\mathrm{C}}_{\uppsi }^{x}\left(a,\tau \right)\right|}^{2}}{{a}^{2}} da d\tau = {\int }_{-\infty }^{\infty }{x(t)}^{2}dt$$

6 $${\pi }^{-1/4}{e}^{i{\omega }_{0}\theta }{e}^{-{\theta }^{2}/2}$$

Since the time series length is finite and needs to maintain the same length to compute the next higher power of two (2, 4, 8, 16, …) to be aligned with the time series, errors occur at the beginning and end of the wavelet power spectrum closer to the edge. To minimize the effect of the errors in the edge points (edge effects), the time series is padded with zeros before performing the wavelet transform and then removing them afterward. The region of the wavelet spectrum that has the edge effect is marked as a cone of influence (COI) and is defined as the e-folding time. The edge effects are dropped by a factor of ${e}^{-2}$, and the power spectrum outside the cone of the influence line is neglected^23,24^. The wavelet power spectrum is normalized by the variance of the time series and then approximated by a Chi-square distribution. The test of significance α = 0.05 is based on the first-order Autoregressive (AR(1)) model, and a new sample is created using the bootstrap technique or by drawing an error from a Gaussian distribution^1,2,28^.

### Wavelet coherence analysis

We can measure the relationships between two time series profiles using wavelet coherence analysis^19^. The power distribution of two time series signals is measured by using wavelet cross spectrum as explained in Eq. (7), where $S$ is a smoothing operator and * is a complex conjugate. The wavelet coherence $({R}^{2})$ is expressed in Eq. (8), and the value of ${R}^{2}\left(a,b\right)$ lies between 0 and 1^17^. It gives the direction and strength of the correlation between two time series by mining the localized frequency events^17,29,30^.

7 $${C}_{xy}\left(a,b\right)=S({C}_{x}^{*}\left(a,b\right){C}_{y}\left(a,b\right))$$

8 $${R}^{2}\left(a,b\right)=\frac{{\left|S({C}_{x}^{*}\left(a,b\right){C}_{y}\left(a,b\right))\right|}^{2}}{{S(\left|{C}_{x}\left(a,b\right)\right|}^{2}).{S(\left|{C}_{y}\left(a,b\right)\right|}^{2})}$$

## Results and discussions

### Periodicity of NO_2_

The results are interpreted based on the seasonal classification of UAE as presented in^31^: Spring (Mar, Apr, and May), Summer (June, July, and Aug), Autumn (Sep, Oct, and Nov) and Winter (Dec, Jan, and Feb). The color bar of the wavelet power spectrum ranges from blue to red, which describes the power intensity from low to high, respectively. The cone of influence is presented in black, and the contour assigns the 5% significance level of the wavelet power spectrum. If the time period intersects with the cone of influence, then it is not considered in the periodicity interpretation, as it might have an edge effect or a high scale value.

#### Abu Dhabi capital region

**Figure 3:**
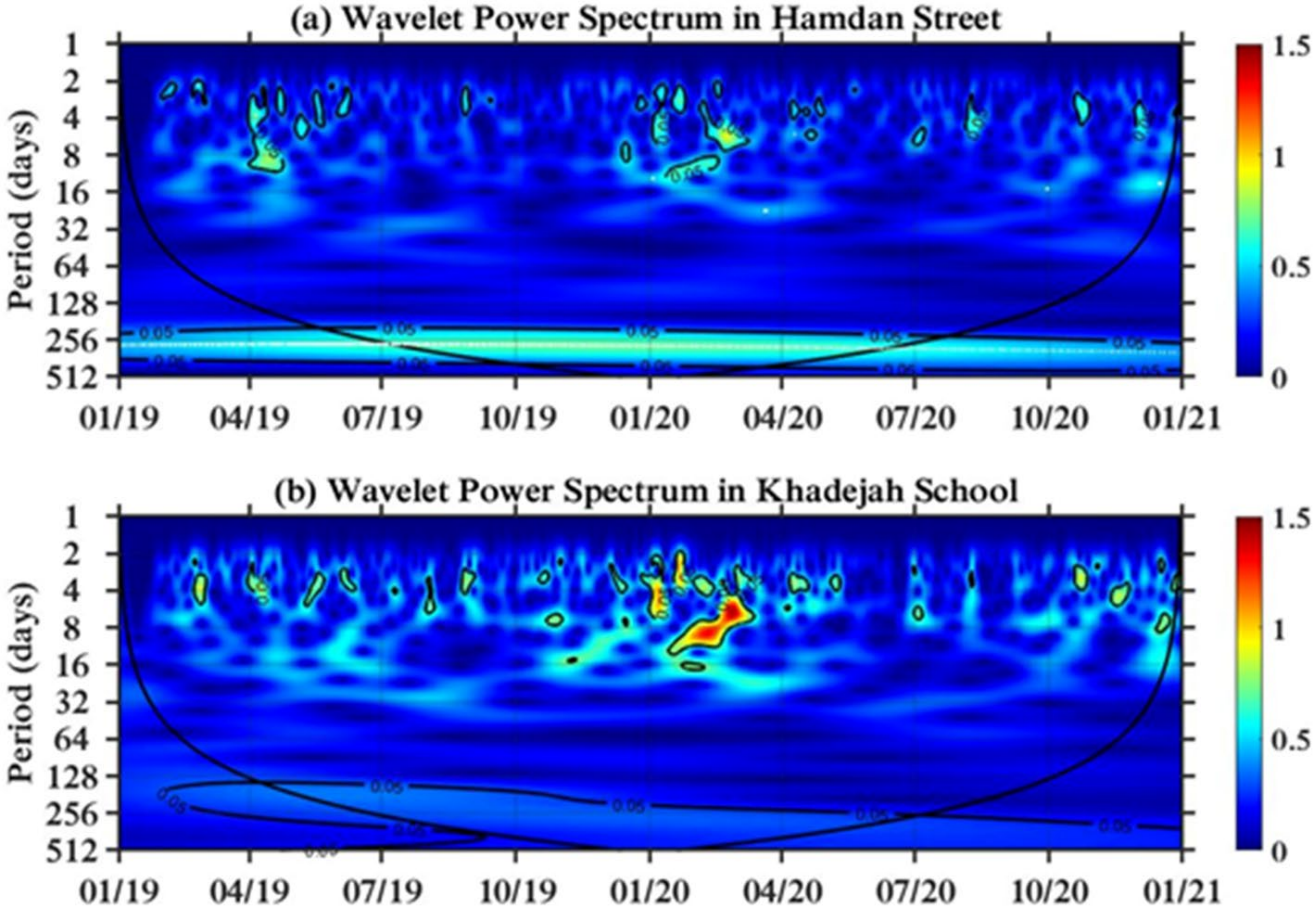
Wavelet power transform in urban areas of the Abu Dhabi capital region: (**a**) Hamdan Street and (**b**) Khadejah School.

The periodicity of the NO_2_ concentration for Hamdan Street differs between 2 and 14 days, as shown in Fig. 3a. The NO_2_ concentration over a period of 14 days appeared in Apr 2019 and from Jan to Feb 2020. During the lockdown period (April–July 2020), the NO_2_ concentration was much lower than that during the same time period in 2019, a result of the COVID-19 pandemic. The highest intensity of the power spectrum occurs in the wintertime between Dec 2019 and Feb 2020. However, the lowest power spectrum is during the summer. The periodicity of NO_2_ for Khadejah School is similar to that for the Hamdan Street ground station, as presented in Fig. 3b, with the highest power spectrum presented during the winter between Dec 2019 and Feb 2020, which is equal to 1.4410 and shown in red. The geographic locations of Hamdan Street and Khadejah School are close to each other, and there are similarities in the periodicity of NO_2_ and the wavelet power spectrum.

**Figure 4:**
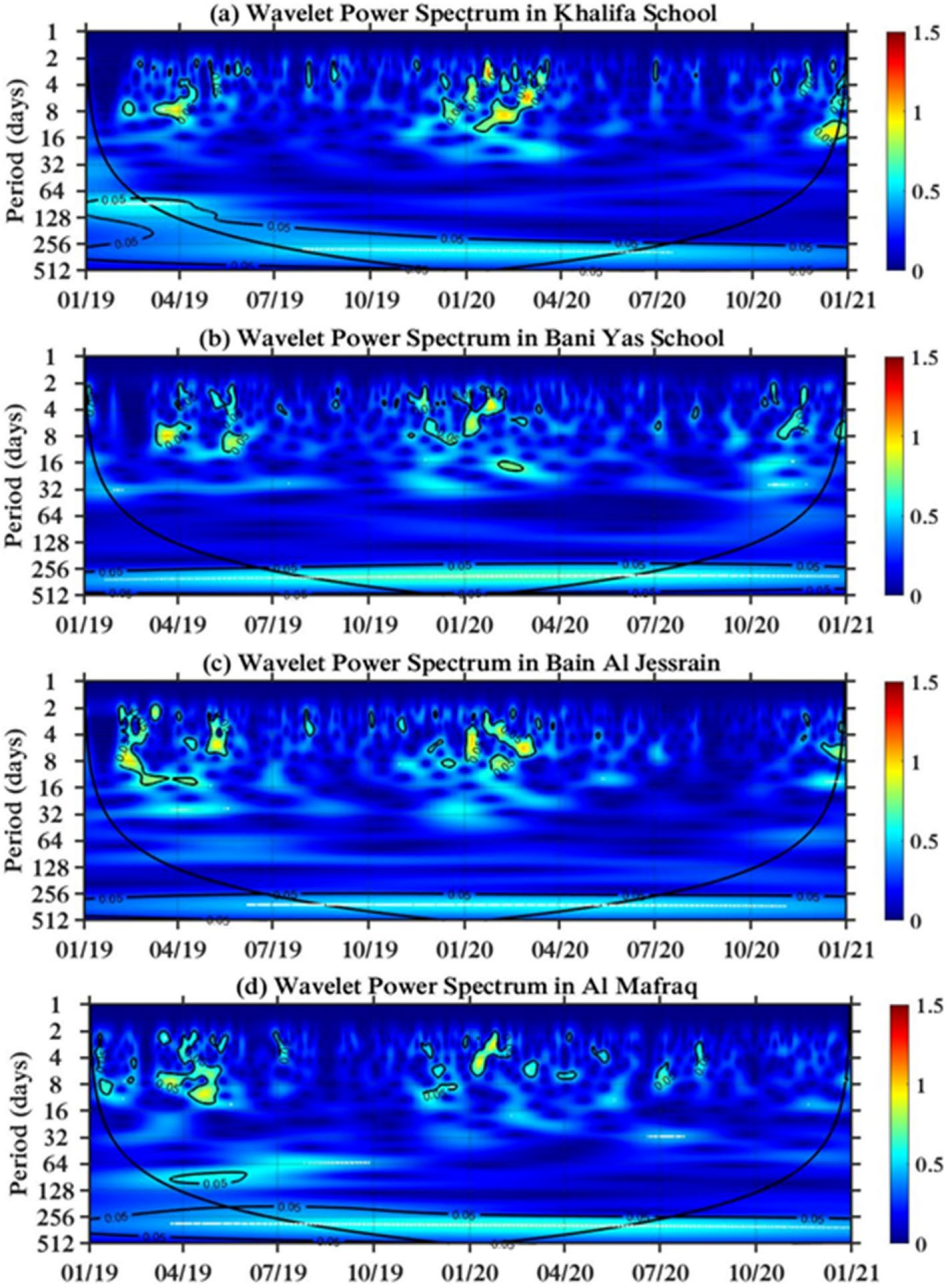
Wavelet power transform in suburban areas of the Abu Dhabi capital region: (**a**) Khalifa School, (**b**) Bani Yas School, (**c**) Bain Al Jessrain, and (**d**) Al Mafraq.

The periodicity of NO_2_ for the Khalifa School is similar to those for Hamdan Street and the Khadejah School, as shown in Fig. 4a. However, in the Bani Yas School in Fig. 4b, the periodicity range extends from two days to over 14 days (almost 16 days), which occurs in the winter months (December 2019 to February 2020), and no periodicity is shown during the lockdown month (April–July 2020). In the Bain Al Jessrain and Al Mafraq, as presented in Fig. 4c,d, NO_2_ is highly concentrated in the winter months, and a reduction in NO_2_ is observed during the lockdown period. However, the highest periodicity is measured in Al Mafraq and lasts between 64 and 128 days, though its intensity is approximately 0.5 that of the higher intensity associated with periodicity ranges of a few weeks.

#### Al Dhafra and Al Ain regions

**Figure 5:**
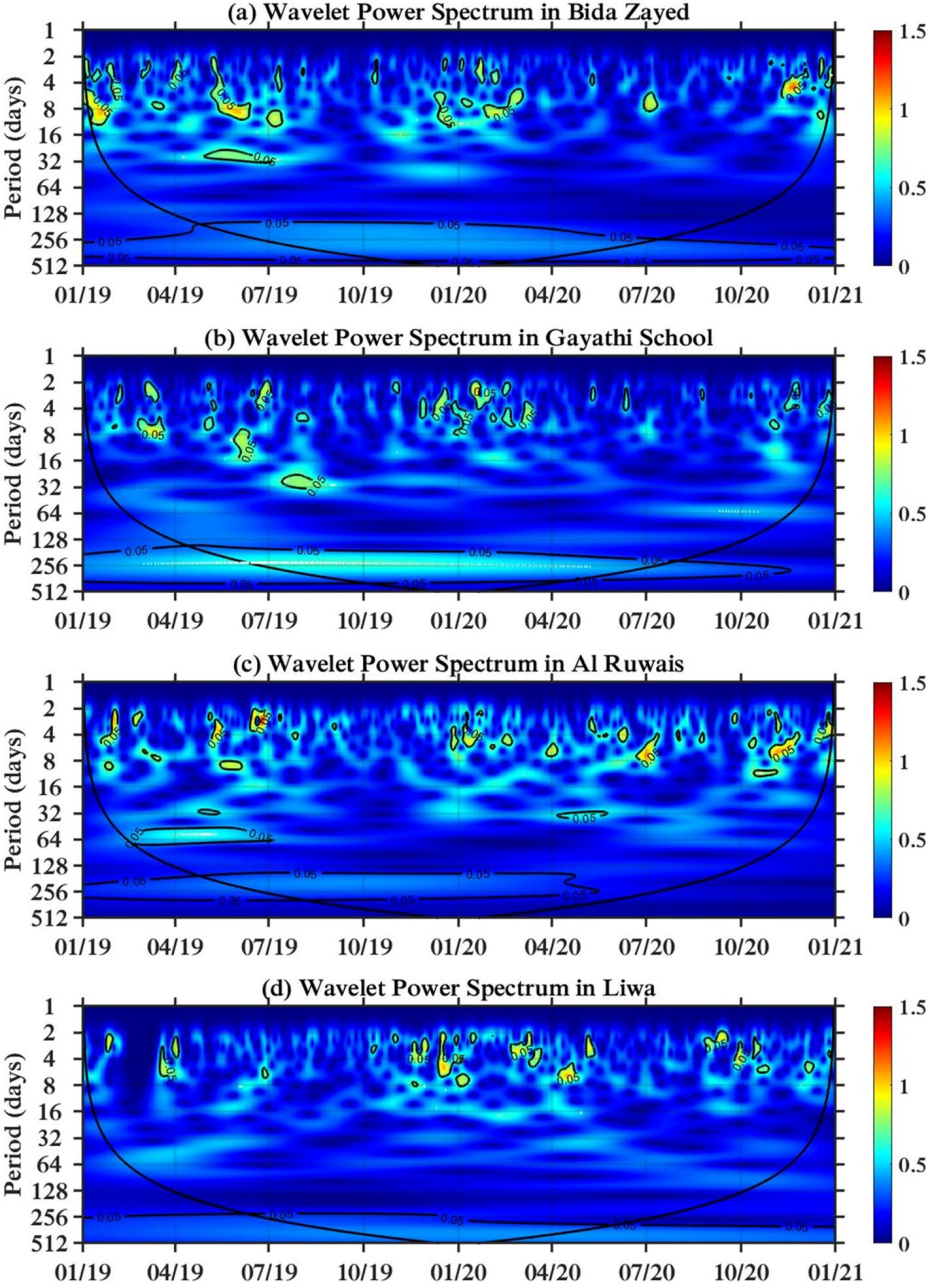
Wavelet power transform in the Al Dhafra region: (**a**) Bida Zayed, (**b**) Gayathi School, (**c**) Al Ruwais, and (**d**) Liwa.

**Figure 6:**
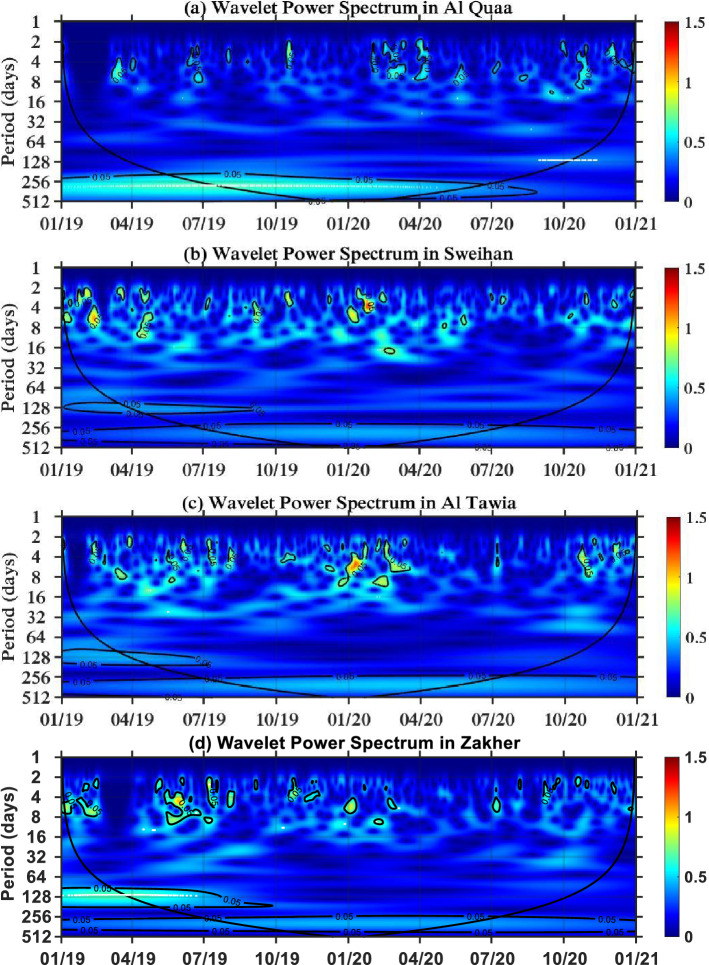
Wavelet power transform in the Al Ain region: (**a**) Al Quaa, (**b**) Sweihan, (**c**) Al Tawia, (**d**) Zakher.

The Al Dhafra region contains the ground stations of Bida Zayed, the Gayathi school, Al Ruwais and Liwa. The periodicity range is higher than that in the Abu Dhabi region. The periodicity of NO_2_ in Bida Zayed and the Gayathi School has a range of up to 32 days. This is noticed in a time range from July 2019 to Sep 2019 in Bida Zayed and from March 2019 to July 2019 in the Gayathi School, as presented in Fig. 5a,b, respectively. However, the periodicity of NO_2_ in Al Ruwais is extended to 64 days in the spring season of 2019, which is less intense than the periodicity that lasts approximately 14 days, as shown Fig. 5c. Two stations in the Al Dhafra Region, which are Bida Zayed and the Gayathi School, shows a reduction in NO_2_ concentrations during the lockdown of the COVID-19 pandemic, whereas there is no noticeable reduction at the Al Ruwais site. However, Liwa is a rural area located in the Al Dhafra region; it was unaffected by the lockdown, yet its periodicity of NO_2_ matches the observations in Al Quaa, which is also a rural area located in the Al Ain region with a range of periodicity from 2 to 8 days. The wavelet power spectra of Liwa and Al Quaa are shown in Figs. 5d and 6a, respectively.

The other stations in the Al Ain Region, such as Sweihan, Al Tawia, and Zakher, have the same periodicity range of 2–14 days, and the concentration of NO_2_ is reduced between April and July 2020 due to the COVID-19 pandemic, as presented in Fig. 6b–d.

### Relationships between station pairs

In addition to the wavelet analysis of individual ground stations, we carried out wavelet coherence analysis on pairs of ground stations. Here, as selected case studies, we show two examples of wavelet coherence analysis for two pairs of remotely located stations.

**Figure 7:**
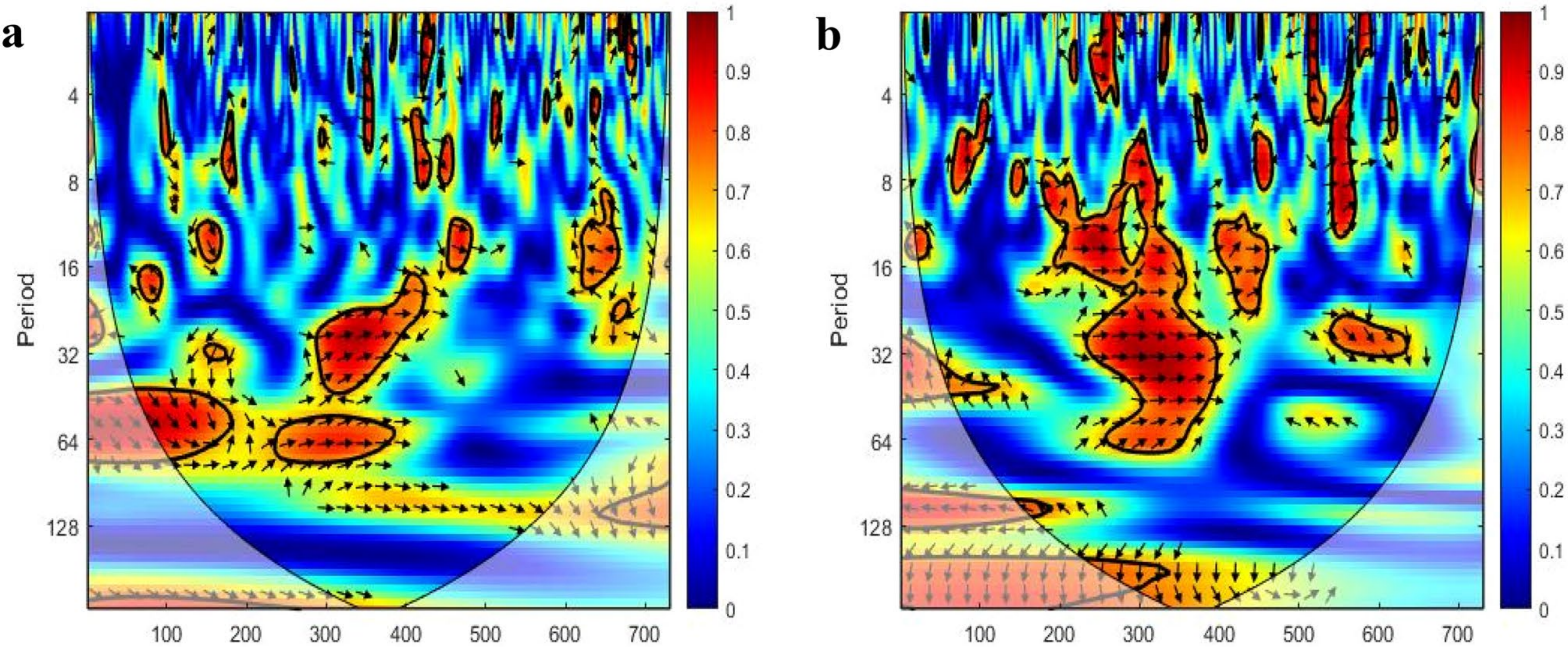
Wavelet coherence: (**a**) wavelet coherence of Liwa vs. Al Quaa ground stations. (**b**) Wavelet coherence of the Al Mafraq vs. Al Ruwais ground stations (the x-axis shows the number of days from the starting date of January 1st, 2019).

First, wavelet coherence of NO_2_ concentrations between the Liwa and Al Quaa ground stations (two rural background stations from the two different regions of Al Dhafra and Al Ain, respectively) is presented in Fig. 7a. The red islands are scattered in the figure. In the frequency band from 16 to 32 days, the correlation of NO_2_ between the two stations is perfect phase, as the arrows point to the right in a time period between October 2019 and March 2020. Additionally, the same case occurs in the red region at a 64-day frequency between August 2019 and January 2020. The other red regions in the band frequency between 8 and 16 present some uncertainties.

As a second example, wavelet coherence of NO_2_ concentrations between the Al Mafraq and Al Ruwais ground stations (two suburban industrial stations from Abu Dhabi city and Al Dhafra regions, respectively) is illustrated in Fig. 7b. The largest red region in the middle of the figure presents between the 8- and 64-day frequency bands during the second half of 2019. The right arrows indicate the perfect correlation between the NO_2_ concentrations at the Al Mafraq and Al Ruwais ground stations during the second half of 2019. The right arrows indicate the perfect correlation between the NO_2_ concentrations at the Al Mafraq and Al Ruwais ground stations. The right upward direction shows the uncertainties, and the right downward arrows specify the leading of the variable^29,30^.

### Discussion of results

The concentration of NO_2_ in rural desert areas such as Liwa and Al Quaa is unaffected by the lockdown period (April–July 2020) that resulted from the COVID-19 pandemic. The other stations in Abu Dhabi, Al Dhafra and Al Ain showed a reduction in NO_2_ during the lockdown. This implies that the NO_2_ concentration generally tends to last longer in the less populated areas than in the more populated areas, probably because of more dynamic atmospheric conditions due to human activities in the latter. This finding is consistent with those of other studies conducted in China^12^, Poland^13^, and India^14^.

The highest concentration of NO_2_ is in the wintertime. This is noticed at all the ground stations when compared to the low concentration in summer. The result agrees with the findings in^7^ for northern Italy with respect to the relation between the concentration of NO_2_ and the seasons. The periodicity of NO_2_ lasted from a few days up to 16 days in most regions. The relatively high intense concentration of NO_2_ occurred at the Khadeja School during winter.

Although the NO_2_ concentrations in Al Mafraq and Hamdan Street fall under one cluster (according to hierarchical clustering analysis), a high correlation is shown between Al Mafraq and Al Ruwais in the second half of 2019, which was the pre-lockdown period (according to wavelet coherence analysis). However, the correlation patterns during the lockdown are less pronounced. This might be because of the stronger effect of COVID-19 in the industrial areas of large cities (e.g., Abu Dhabi, where Al Mafraq is located) compared to the industries in small towns (e.g., Al Ruwais). However, the concentrations in Liwa and Al Quaa, both of which are rural deserts, exhibit good correlation in wavelet coherence analysis and lie under one cluster in clustering analysis.

## Conclusion

In this study, we employed wavelet spectral analysis (particularly Morlet continuous wavelet transform) for the NO_2_ concentration levels of the United Arab Emirates (UAE); these data came from fourteen ground stations. To the best of our knowledge, this study is the first of its kind to use wavelet analysis on a greenhouse gas in the UAE. We studied the periodicity of NO_2_ levels over the period of 2019–2020, which included the COVID-19 lockdown period. Our study found that the lockdown did affect the atmospheric concentration of NO_2_, especially in urban and industrial areas. We also found a generally seasonal pattern of NO_2_ in the UAE. In addition, we show a good correlation in the NO_2_ concentration profiles of stations located in areas of similar land uses even though these stations are geographically distanced.

The concentration of NO_2_ in the atmosphere fluctuates according to human activities and the presence of factories in industrial areas. Tracking the variability of NO_2_ concentrations needs to be studied accordingly with population intensity and other environmental factors, such as temperature, which affects the movement of NO_2_ in the atmosphere. It is vital to study the concentration of NO_2_ via many other natural trappers, such as water and sand, because NO_2_ can change from one location to another. As a future work, we plan to study the environmental concentration of NO_2_ in UAE using X-ray powder diffraction (XRD) for environmental samples, such as sand^32^.

## Data Availability

The ground station data used in the study to support findings were made available by Environment Agency—Abu Dhabi specifically for the study under a license agreement. Hence, the data are not publicly available and must be requested directly from the Environment Agency—Abu Dhabi by writing to customerhappiness@ead.gov.ae.
